# Spatial distribution of deaths due to Alzheimer's disease in the state of São Paulo, Brazil

**DOI:** 10.1590/1516-3180.2014.1324610

**Published:** 2014-05-20

**Authors:** Milena Cristina da Silva Almeida, Camila de Moraes Santos Gomes, Luiz Fernando Costa Nascimento

**Affiliations:** I Medical Student, Department of Medicine, Universidade de Taubaté (UNITAU), Taubaté, São Paulo, Brazil; II PhD. Assistant Professor, Department of Medicine, Universidade de Taubaté (UNITAU), Taubaté, São Paulo, Brazil

**Keywords:** Alzheimer disease, Geographic information systems, Residence characteristics, Mortality, Health of the elderly, Doença de Alzheimer, Sistemas de informação geográfica, Distribuição espacial da população, Mortalidade, Saúde do idoso

## Abstract

**CONTEXT AND OBJECTIVE:**

Alzheimer's disease is a common cause of dementia and identifying possible spatial patterns of mortality due to this disease may enable preventive actions. The objective of this study was to identify spatial distribution patterns of mortality due to Alzheimer's disease in the state of São Paulo.

**DESIGN AND SETTING:**

Ecological and exploratory study conducted in all municipalities in the state of São Paulo.

**METHODS:**

Data on Alzheimer's disease mortality in the state of São Paulo between 2004 and 2009 were obtained from DATASUS (the Department of Informatics in the Brazilian Ministry of Health). Death rates per 100,000 inhabitants were then calculated and spatial analysis was performed by constructing a death rate map, global Moran index and local Moran index, which were used to obtain the Moran map. The kernel technique was also applied. The Terra View 4.0.0 software was used.

**RESULTS:**

13,030 deaths due to Alzheimer were reported in the state of São Paulo (rate of 5.33 deaths/100,000 inhabitants). São José do Rio Preto, Ribeirão Preto, Bauru and Araçatuba had higher rates. The Moran index was I = 0.085 (P < 0.002). The Moran map identified 42 municipalities that merit intervention and the kernel estimator identified a high density of deaths in the northwestern region of the state.

**CONCLUSION:**

Higher densities of deaths due to Alzheimer were concentrated more to the north and northwest of the state of São Paulo. It was possible to identify municipalities that have priority for interventions to reduce the death rates due to this disease.

## Abstract (pt)

**CONTEXTO E OBJETIVO:**

A doença de Alzheimer é uma frequente causa de demência e a identificação de possíveis padrões espaciais da mortalidade por essa doença pode permitir ações preventivas. O objetivo do trabalho foi identificar padrões espaciais da distribuição da mortalidade por doença de Alzheimer no estado de São Paulo.

**DESENHO E LOCAL DE ESTUDO:**

Estudo ecológico e exploratório realizado em todos os municípios do estado de São Paulo.

**MÉTODOS:**

Foram obtidos, do DATASUS (Departamento de Informática do SUS, Sistema Único de Saúde), dados sobre mortalidade por doença de Alzheimer, no estado de São Paulo entre 2004 e 2009. Foi calculada a taxa de óbitos por 100.000 habitantes e realizou-se a análise espacial, por meio da construção do mapa da taxa de óbitos, do índice global de Moran e do índice local de Moran, utilizado para a obtenção do Moran Map. Aplicou-se também a técnica de kernel. Foi utilizado o programa TerraView 4.0.0.

**RESULTADOS:**

Foram notificados 13.030 óbitos por Alzheimer no estado de São Paulo (taxa de 5,33 óbitos/100.000 habitantes). São José do Rio Preto, Ribeirão Preto, Bauru e Araçatuba apresentaram altas taxas. O índice de Moran encontrado foi I = 0.085 (P < 0,002). O Moran Map identificou 42 municípios que merecem intervenção e o estimador de kernel identificou alta densidade de óbitos na região noroeste do estado.

**CONCLUSÕES:**

Maiores densidades de óbitos por Alzheimer se concentraram mais ao norte e noroeste do estado de São Paulo. Foi possível identificar municípios que têm prioridade de intervenção para diminuição das taxas de óbitos por esta doença.

## INTRODUCTION

Alzheimer's disease (AD), a common cause of dementia, is characterized by progressive neurodegenerative disorders with progressive loss of memory and behavioral disorders that diminish individuals' quality of life. Extracellular amyloid plaque and intraneuronal neurofibrillary tangles accumulate.^1^ ^,^ ^2^

The prevalence of dementia in several regions of the world ranges from 1.17% in the population between 65 and 69 years of age to over 50% of the population over 95 years of age.^3 ^A Brazilian study identified a prevalence of 7.1% for any type of dementia in the elderly population, and 54% of the cases were AD.^4^ The female sex has been identified as the one most affected by AD, but it is not yet known whether this is due to a genetic factor or because women are more prevalent in the elderly population.^3^ ^,^ ^4^ A review of the Brazilian literature found a prevalence of dementia ranging from 5.1% to 19% in São Paulo, which may indicate that São Paulo has a prevalence closer to that of developed countries than to the prevalence in other regions of Brazil.^5^

With the progressive aging of the population, and the estimate that in 2025 there will be 34 million people over 60 years of age in Brazil,^6^ the incidence of AD is increasing every year and has become a public health problem.^7^ The main cause of the disease is genetic, but states of toxicity to infectious agents, aluminum, free oxygen radicals and neurotoxic amino acids have already been shown to be factors implicated.^8^ ^,^ ^9^

Mapping of disease has becomes an important public health tool, in relation to both diagnosis and planning. Spatial analysis enables implementation of health programs that include several municipalities or regions of a state and thus has also become an important tool for public health diagnosis and planning.^10^ ^,^ ^11^ The rapid development of technologies for data analysis covering geographical space has provided innovative possibilities for studying the health situation and its trends, thus allowing better understanding of the socioeconomic and environmental factors.^12^

An overview of environmental conditions and population health can be provided by maps that enable observation of the spatial distribution of risk exposures and health problems.^13^ Thematic maps can be used with an exploratory approach, since they show the location of the event and unknown etiological factors that hypothetically may be used to point out links between the event and its causes.^14^

## OBJECTIVE

To identify spatial distribution patterns of mortality rates due to Alzheimer's disease in the state of São Paulo over the period from 2004 to 2009.

## METHODS

### Type of study

This was an ecological and exploratory study using data on mortality due to Alzheimer's disease obtained from the Department of Informatics of the Ministry of Health (DATASUS)^15^ for the period between January 1, 2004, and December 31, 2009.

### Setting

This study was conducted in 645 municipalities in the state of São Paulo, Brazil, which has more than 40 million inhabitants.

### Sample 

We used data on mortality due to Alzheimer's disease obtained from the DATASUS Mortality Information System (SIM). These data were coded in accordance with ICD-10, in the G30 group of causes (061 Alzheimer Disease).

### Procedures

Initially, a database was constructed with all the cases of mortality due to Alzheimer's disease. These cases were analyzed as the rate per 100,000 inhabitants. This spreadsheet, in DBF format, was imported by means of Terra View 4.0.0, which is a public-access software developed by INPE (http://www.dpi.inpe.br/). The spatial analysis was performed on a digital database of the municipalities of the state of São Paulo that was obtained from the Brazilian Institute of Geography and Statistics (IBGE).

### Statistical analysis

The analysis estimated the spatial autocorrelation coefficient of events by means of the global Moran index (I). The global Moran index establishes the degree to which the value of a variable in a region (a city, for example) is similar to the values for this same variable in neighboring regions. The values of this index can range between -1 and +1. The closer to +1 that the index is, the greater the similarity is between that region and its neighbors, with regard to the variable studied. On the other hand, the closer to -1 that it is, the more different (less similar) the values of this region are, in relation to neighboring regions. If the value is zero, this indicates spatial independence.

We also used the local Moran index (LISA), which determines the dependence of the local data in relation to their neighbors. This index made it possible to identify the groups (clusters) and subregions where the spatial dependence occurs. It was possible to graphically analyze the degree of similarity between the neighbors, thus indicating areas of high priority for attention, through the Moran scatter plot. The quadrants Q1 and Q2 indicated points of positive spatial association, meaning that a place had neighbors with similar values. The quadrants Q3 and Q4 showed points of negative spatial association, such that a place had neighbors with different values. The municipalities targeted for intervention would be those located in Q1. The Moran map was constructed, which was the representation of the local Moran index.

Only the municipalities for which the LISA values were considered significant were highlighted, with assigned values of one. The other areas did not have significance. In addition to these indices, we used the statistical technique of kernel smoothing, which identified the areas of highest density of cases.

## RESULTS

Between 2004 and 2009, 13,030 deaths due to Alzheimer's disease were reported in the state of São Paulo, representing a rate of 5.33 deaths per 100,000 inhabitants. 

**Figure 1:**
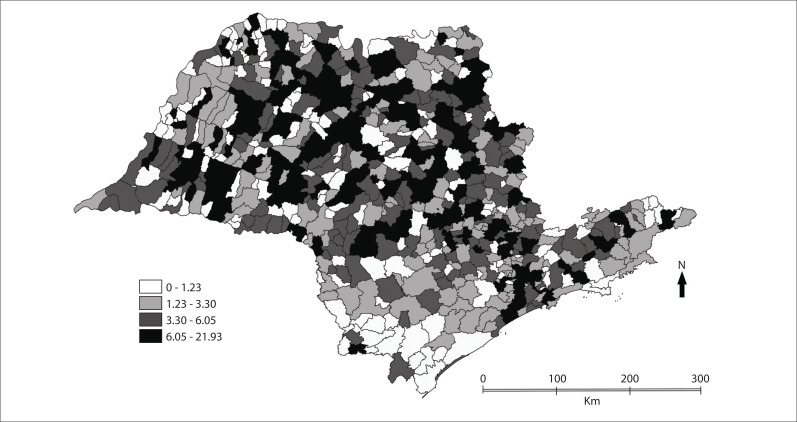
Thematic map of Alzheimer's disease deaths per 100,000 inhabitants, state of São Paulo, 2004-2009.

Figure 1 shows the spatial distribution of mortality due to Alzheimer's disease. It can be seen that in the north and northwest of the state, there was greater concentration of high rates of deaths per 100,000 inhabitants. The cities of São José do Rio Preto, Ribeirão Preto, Bauru and Araçatuba had rates of between 7.56 and 10.40 deaths/100,000 inhabitants. In the central region of the state, the municipalities highlighted were Rio Claro, Botucatu, Piracicaba and Limeira, which showed rates of between 6.90 and 12.00/100,000 inhabitants. In the southeastern and coastal regions, the municipalities of São Caetano do Sul, with a rate of 19.42, and Santos, with a rate of 13.39, had the highest rates and were the only ones highlighted. In the southern region of the state, low rates of deaths due to AD predominated.

**Figure 2:**
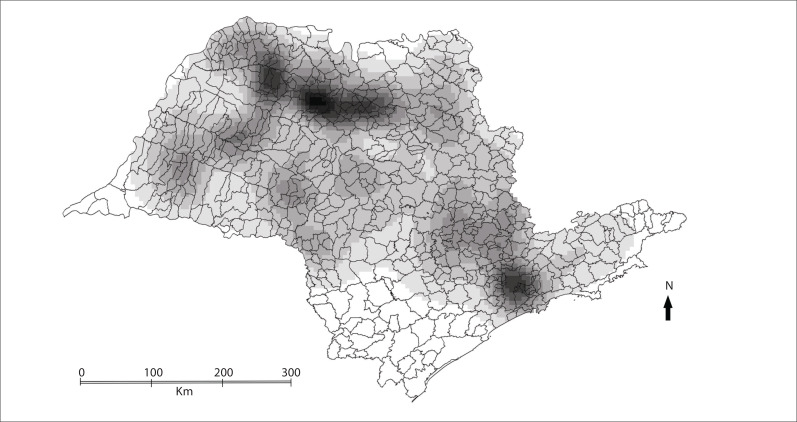
Thematic map of kernel estimator for death rate per 100,000 inhabitants, due Alzheimer's disease, state of São Paulo, 2004-2009.

**Figure 3:**
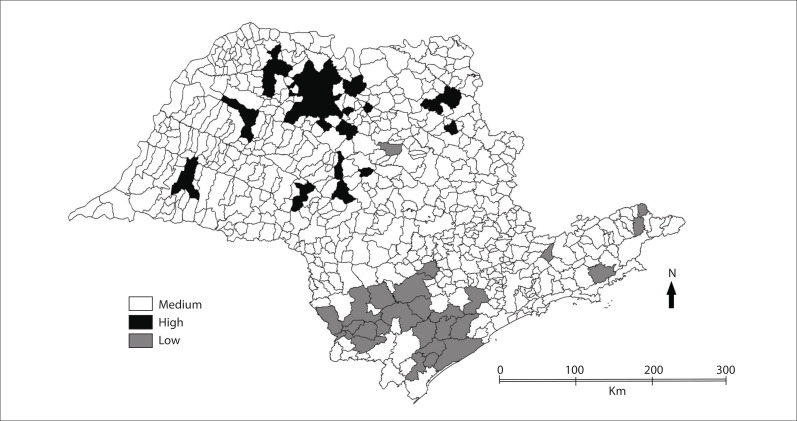
Thematic map for Moran map, for death rate due to Alzheimer's disease, state of São Paulo, 2004-2009.

The spatial analysis results showed that the global Moran index (I) was 0.09 (P < 0.01), thus showing a positive spatial autocorrelation for deaths due to AD. Figure 2 shows the map of the kernel death rate per 100,000 inhabitants, clearly indicating a hot spot in the northern region of the state, around the city of São José do Rio Preto. Figure 3 shows the Moran map, on which the municipalities with high priority for intervention are indicated. These municipalities need to be better investigated, since they have high rates and are also surrounded by other municipalities with high rates. 

The municipalities indicated with lighter colors are cities with low priority for attention, because they showed low rates. Some of these municipalities are in the south, toward the state of Paraná.

## DISCUSSION

This study identified municipalities with high rates of deaths due to AD. It was the first, as far as we know, to use spatial analysis to identify clusters of municipalities with high rates of this outcome, in the state of São Paulo.

The number of cases of AD has increased because of the ageing of the population, given that this disease affects the elderly, and also because better diagnoses have been accomplished. The advances in diagnosing AD have come from more elaborate clinical, laboratory and imaging examinations and from better understanding of AD and other types of dementia, thus enabling characterization in terms of patient history, physical examinations and application of tests on cognition, memory, attention, executive functions and other tests.^16^ However, knowledge about the disease and these advances in diagnosis are not yet available for all municipalities, and one limitation of the present study is the possibility of wrong diagnosis of the disease, especially in poorer districts, along with underreporting.

Although AD is a genetic disease, observation of the spatial distribution seen in this study makes it possible to hypothesize that other predisposing factors may be involved, such as environmental factors. As an example, given that sugar cane, oranges and lemons are widely cultivated in this region, use of pesticides on these crops could be one of these factors. A cohort study conducted on an elderly population in France showed that occupational exposure to pesticides suggests that neurological disability will be present. The study showed that occupational exposure to any pesticide was associated with a twofold increase in the risk of development of AD.^17^ Chronic exposure, even at low doses, is associated with neuropsychiatric symptoms, and therefore an association between chronic exposure to pesticides and development of neurodegenerative diseases should be taken into consideration.^18^

Another possibility would be exposure to magnetic fields. A relationship between exposure to high voltage lines and magnetic fields and development of cancers such as leukemia has been suggested in several studies,^19^ ^,^ ^20^ and this is also a possible factor in the development of AD. 

The presence of highways such as Washington Luiz (SP-310), Transbrasil (BR-153), Marechal Rondon (SP-300) and Euclides da Cunha (SP-320) in the north and northwest of the state of São Paulo may cause air pollution, which may be reflected in development of diseases such as respiratory diseases, thus perhaps forming another environmental factor that contributes towards development of AD.

As has been pointed out in other studies,^8^ ^,^ ^9^ there may be an association between exposure to aluminum and AD. Aluminum and its derivatives are used in water treatment, food processing, pharmaceutical manufacture and foundries, thus leading to occasional human exposure to this metal.

This study has limitations such as those that are intrinsic to ecological studies. Moreover, there may have been errors in filling out the primary cause on the death certificate, thus leading to underreporting. Moreover, it is also possible that some people died due to Alzheimer's disease as a secondary cause, but were not identified, thus compromising the total number of events. 

Demographic parameters were not considered in this study because they are not available through DATASUS. The IPRS, i.e. the Index of Social Responsibility, created by the State Data Analysis System (SEADE), which takes into account wealth, longevity and schooling as parameters for assessing the situation of each municipality, could have been taken into consideration. However, the main objective here was to identify the spatial pattern of deaths due to Alzheimer's disease.

The strength of this study is that it indicates municipalities in which health managers can implement interventions. Even though the high rates in these regions may have been due to better diagnosis, these are still municipalities in which investigation of occurrences of Alzheimer's disease are merited. These interventions will allow future comparisons with current data. 

Further research will be based on the present study, including sociodemographic data. This study fills a gap in the literature on Alzheimer's disease and it is difficult to compare it with other Brazilian data.

## CONCLUSIONS

Between 2004 and 2009, the mortality rates due to Alzheimer's disease were higher in the north and northwest of the state of São Paulo. From observation on the distribution of deaths due to Alzheimer's disease, it is concluded that spatial analysis is a relevant technique for studying this disease, because it enables identification of clusters of cities, thereby making better focused actions possible.
